# Reply to M Mindrum and J Moore et al

**DOI:** 10.1093/cdn/nzac029

**Published:** 2022-05-27

**Authors:** David S Ludwig, Nicholas G Norwitz, David Feldman, Adrian Soto-Mota, Tro Kalayjian

**Affiliations:** Harvard Medical School, Boston, MA, USA; Harvard Medical School, Boston, MA, USA; Citizen Science Foundation, Las Vegas, NV, USA; Metabolic Diseases Research Unit, National Institute for Medical Sciences and Nutrition Salvador Zubiran, Tlalpan, Mexico; Yale New Haven Health System, New Haven, CT, USA

Dear Editor:

We appreciate the opportunity to respond to the letters of Mindrum and of Moore et al. about our study on a novel “lean mass hyper-responder” (LMHR) phenotype (1).

We agree with Mindrum's call for more research, including long-term prospective studies with cardiovascular imaging. We respectfully disagree with his concern that patients may be misled regarding the safety of high LDL cholesterol (LDLc). We aimed to be cautious in terminology and conclusions. The first sentence of the paper states that elevated LDLc is “an important risk factor for atherosclerotic cardiovascular disease (ASCVD).” The last sentence of the paper emphasizes that “This study should not be interpreted as implying cardiovascular safety of the LMHR phenotype.” In between, we use other terminology (e.g., “extreme” and “severe”) to characterize the nature of the LDLc elevations observed. Furthermore, our case series suggests a simple dietary intervention to ameliorate the LDLc elevation in this phenotype.

Mindrum writes that “with all other cardiovascular risks being equal, the null hypothesis is that the higher the apo-B, the higher the cardiovascular risk.” However, all other risks may not be equal within the context of a low-carbohydrate diet. As reviewed by Libby (2), high triglycerides (TGs), low HDL cholesterol (HDLc), and small LDL particle size now comprise the dominant dyslipidemia in ASCVD. Among dietary options, carbohydrate restriction is more effective at targeting these and associated components of the metabolic syndrome than fat restriction, even with control for calorie intake in randomized controlled trials (3, 4). Of note, participants in our study had an exceptionally low TG:HDLc ratio, a marker of insulin sensitivity. Thus, the increase in LDLc is accompanied by improvement in metabolic syndrome components; the latter is more difficult to treat pharmacologically. Nevertheless, we acknowledge that extreme elevations of LDLc with a low-carbohydrate diet may confer major ASCVD risk, despite any associated benefits for metabolic syndrome, in the small minority of people with this response.

Clearly, the pathophysiology of atherosclerosis is complex, with many genetic and other factors likely interacting to determine the extent to which cholesterol-containing apoB particles cause harm. One possibility is that the relation between LDL particle number and ASCVD has a similar slope throughout the population, although at different levels of absolute risk based on the presence of other risk factors. Another possibility is that LDL particles are inherently less dangerous on a background of low insulin resistance, chronic inflammation, and oxidative stress. This question, as it pertains to the LMHR phenotype, will require long-term prospective studies.

With regard to Mindrum's other points, we considered SGLT2 inhibitors to highlight potentially shared mechanisms related to a shift from carbohydrate to fat metabolism, not to suggest that this drug raises LDLc to the degree observed in the LMHR phenotype. Finally, Mindrum states that we failed to reference randomized controlled trial data in young, lean women with markedly elevated apoB following a carbohydrate-restricted diet. This study by Buren et al. (5) was the second reference in our paper.

Moore et al. advance 4 methods-related and 4 interpretative criticisms of our study, but these involve some misdirection. We address each of these in a point-by-point fashion as follows:

*Methods Point 1*. Covariates such as diet were not included in the statistical models and selection bias exists. These issues were explicitly and extensively considered in our paper, the intent of which was to present preliminary descriptive data on a previously unrecognized diet–phenotype interaction. We acknowledged the potential selection bias in our sample and recognized that additional research will be needed to examine the generalizability and clinical translatability of our findings.

*Methods Point 2*. Use of the TG:HDLc ratio requires justification. High TGs and low HDLc comprise core components of the metabolic syndrome, originally described as syndrome X (or the insulin resistance syndrome) by Reaven in the 1980s (6). As considered in our paper, a high TG:HDLc ratio and increased small LDL particle concentration characterize the dominant atherogenic dyslipidemia today (2), for which carbohydrate restriction holds special promise (3, 4).

*Methods Point 3*. The phenotype should be renamed “normal BMI hyper-responders” because BMI does not measure lean mass or body fat. As considered in the manuscript, the term LMHR is historical, proposed in 2017 by a coauthor (DF) based on theoretical considerations. The accuracy of this name could be reconsidered as additional mechanistic data accrue. With regard to their characterization of 1 patient in our case series (#2) as “not lean for a female,” nationally representative data indicate that 22.5% body fat corresponds to a BMI (in kg/m^2^) <18.5 for a non-Hispanic White woman aged 49 y (7).

*Methods Point 4*. Prior LDLc was omitted from the statistical models; the appropriateness of linear regression for TG:HDLc is questionable. Adjustment for prior LDLc does not materially alter the associations involving TG:HDLc (*P* = 1.96 × 10^–5^ in model 2 after adjustment) and BMI (*P* = 2.78 × 10^–8^ in model 3 after adjustment). It is unsurprising that, in their reanalysis of the decision tree, “the algorithm frequently selected prior LDLc,” in view of the strong intraindividual correlation in repeated measures of LDLc. Furthermore, as depicted in **Figure 1**, the most likely distribution of the residuals is Gaussian. Indeed, the residual's mean is quite close to zero (–3.7 × 10^–15^), the variance inflation factor of both TG:HDLc and BMI is well below 5, and Durbin Watson's test show that autocorrelation is unlikely.

*Interpretations Point 1*. TG:HDLc ratio and BMI account for only a small proportion of the variance in LDLc change. This criticism could be considered a “red herring” fallacy. The aim of our paper is to describe a novel diet–phenotype interaction characterizing a small proportion of the population, not to generate models explaining all possible sources of heterogeneity. Furthermore, it is unsurprising that their alternative model with baseline LDLc would have a nominally higher *R*-squared, as considered above.

*Interpretations Point 2*. The highest BMI quartile experienced an LDLc elevation associated with increased cardiovascular disease risk. Because our respondents were much leaner than the general population, as stated in our paper, “we would expect an even smaller increase in LDLc among individuals with high BMI as compared with the median LDLc increase observed among respondents in the highest BMI” quantiles. Indeed, LDLc often does not increase with carbohydrate restriction in studies of participants with obesity and type 2 diabetes, as reviewed in our paper. We acknowledged that the LDLc elevation in this phenotype may confer significant cardiovascular risk, and further research will be needed to address this possibility.

*Interpretations Point 3*. A study showing no increase in LDLc concentration on a low-carbohydrate diet was high in cheese (8). Moore et al. do not cite a reference for their assertion that cheese protects against LDLc elevation. In any event, the cited study included a wide range of foods representative of prevailing consumption patterns, and the saturated fat content derived from a variety of sources, not primarily cheese (8, 9).

*Interpretations Point 4*. The directed acyclic graph (DAG) is problematic. In online supplementary material, we presented a DAG to explore alternative relations and to argue against a major causal role of saturated fat consumption. We do not intend this DAG to be a precise causal model of the LMHR phenotype, we present low-carbohydrate intake first as it was an a priori inclusion criterion for study participants, and we agree that other DAGs could be constructed.

**FIGURE 1:**
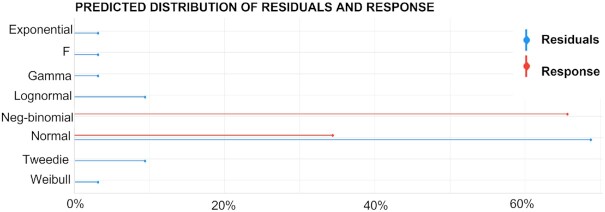
Residuals distribution in model 3 (LDLc change regressed to TG:HDLc ratio and BMI). The appropriateness of linear regression can be corroborated with the R code *plot(check_distribution(m3))/mean(m3$residuals)/vif(m3)/durbinWatsonTest(m3)*. After evaluating 7 possibilities, the most likely distribution of the residuals is normal. The plot was produced with the function *performance::check_distribution*. (Response is LDLc change.) HDLc, HDL cholesterol; LDLc, LDL cholesterol; TG, triglyceride.

## Notes

The authors received no specific funding for this work.

Author disclosures: NGN is coauthor of a Mediterranean low-carbohydrate diet cookbook; he donates all royalty payments to nutrition research and education. DF receives financial contributions from membership (e.g., through Patreon) for continued research and is a partner in Own Your Labs LLC, with all proceeds contributed to the Citizen Science Foundation. TK is an unpaid member of the Board of Directors of Society of Metabolic Health Practitioners and a producer of podcasts on health and nutrition, with all proceeds donated to humanitarian charities, and his spouse has ownership interest in a food company. DSL received royalties for books that recommend a carbohydrate-modified diet, and his spouse owns a nutrition education and consulting business. AS-M reports no conflicts of interest.

Author contributions—DSL and NGN drafted the initial reply. All authors provided input and editorial comments.
